# Feasibility of an exergaming training program in type 2 diabetes mellitus: A mixed method study

**DOI:** 10.1177/20552076241285090

**Published:** 2024-10-14

**Authors:** Nikola Savic, Heidi Petry, Eling D. de Bruin, Roger Lehmann, Patrick Eggenberger, Manuela Adcock, Ruth Hirschmann, Ruud H. Knols

**Affiliations:** 1 Institute of Human Movement Sciences and Sport, Department of Health Sciences and Technology, 27219 ETH Zurich, Zurich, Switzerland; 2 Directorate of Research and Education, Physiotherapy and Occupational Therapy Research Center, 27243 University Hospital Zurich, Zurich, Switzerland; 3 Center for Clinical Nursing Science, 27243 University Hospital Zurich, Zurich, Switzerland; 4 Division of Physiotherapy, Department of Neurobiology, Care Sciences and Society, Karolinska Institute, Stockholm, Sweden; 5 Department of Health, OST-Eastern Swiss University of Applied Sciences, St Gallen, Switzerland; 6 Department of Endocrinology and Diabetology, University Hospital Zurich, Zurich, Switzerland

**Keywords:** Exergaming, feasibility, older adults, heart rate variability, cognitive function, cognitive-motor training, diabetes, physical activity

## Abstract

**Introduction:**

Individuals with Type 2 Diabetes Mellitus may benefit from exergaming training. Exergaming, technology-driven physical activities requiring participants to be physically active or exercise to play the game, allows combining cognitive with motor training. This trial aimed to primarily evaluate the feasibility of an exergame-based training protocol. Secondarily, possible effects on physical, functional, and patient-reported outcomes were explored.

**Methods:**

Type 2 diabetic individuals performed an exergaming protocol on a pressure sensitive platform. After a 6-week control period, training was administered 2–3 times weekly for another six weeks for 30–60 minutes per session. Outcome variables were assessed during baseline (T0), pre-intervention (T1) and twice at postintervention (T1 and T2). An interview after completion ended the study program. Feasibility was determined by recruitment, adherence, compliance, attrition rates, motivation, satisfaction, and technology acceptance.

**Results:**

Eleven of 13 participants completed the study protocol. The feasibility criteria adherence-mandatory (86.4%), adherence-voluntary (70.2%), compliance (99.7%), attrition (15.4%) rate, motivation (82%), satisfaction (80%), and technology acceptance (62.5%) were all deemed acceptable, except for the recruitment rate (13.7%). There were inconsistent effects on functional outcomes, appraisal of diabetes, and health-related quality of life. Qualitative patient-reported experience was overall positive, which is in line with the quantitative results.

**Conclusion:**

The exergame-based training program is feasible and safe and type 2 diabetic participants’ acceptance of this approach was high, although the recruitment procedure needs minor changes. Furthermore, results were obtained that might be useful in selecting appropriate assessments and sample sizes in future trials.

## Introduction

Type 2 diabetes mellitus (T2DM), with 537 million adults affected in 2021, is one of the most prominent emerging public health concerns worldwide strongly contributing to the global burden of the disease.^1,2^ Individuals with T2DM are at elevated risk for microvascular (e.g., neuropathy, nephropathy, and retinopathy) and macrovascular (e.g., high blood pressure, ischemic heart disease, and stroke) consequences.^3,4^ Furthermore, T2DM is a factor leading to a twofold increase in risk for cognitive decline.^4^ According to accumulated evidence, cognitive decline is linked with altered heart rate variability (HRV) indices and affected persons show lowered levels in both sympathetic and parasympathetic nervous system-related HRV indices, as well as global HRV.^5^ It is hypothesized that the main cognitive domain associated with lowered HRV indices includes executive functioning (planning, coordinating, sequencing, and monitoring of cognitive operations).^6^ The deleterious effects of altered glucose metabolism on HRV can both explain this deterioration and the vascular brain damage. This deterioration can, in turn, lead to a worsened self-care, lower performance in activities of daily living, loss of quality of life, and finally institutionalization.^5,7^

The worldwide increasing burden of T2DM is mainly attributable to lifestyle factors such as but not limited to high sugar-sweetened beverages, red meat intake, obesity, high fasting plasma glucose levels, metabolic syndrome, and sedentary behavior.^1,8^ Regarding the latter, there is a strong body of evidence for small-to-moderate effects of conventional exercise on physical and psychosocial outcomes when combined with dietary modifications and anti-diabetic medication.^9,10^ Nevertheless, despite multiple health benefits of physical activity (PA), adherence rates to exercise prescriptions remain low.^11,12^ Main reasons are lack of enjoyment and motivation, absence of social support, disease-related implications such as fatigue or pain, and the unattractive and repetitive nature of the PA programming.^13,14^

There are indications, that the use of technology to monitor and offer PA may positively effect on adherence rates.^15^ Such innovative approaches to PA promotion have the potential to advance treatments through the design of new training devices and strategies that are more motivating. Technology-based approaches also offer the possibility of combining cognitive and motor training while playing interactive video games.^13,16^ Gamification of exercise is called “exergaming,” a portmanteau of “exercise” and “gaming,” and is generally safe, feasible, enjoyable, and motivating. In the context of technology-based approaches, interactive simulations are created with computer hardware and software to present users with opportunities to engage in environments that appear and feel like real-world objects and events while using 2D- or 3D-platforms. It can be interfaced with a head-mounted device, television screen or a mobile app and, therefore, can be stationary or portable. It increases engagement and can augment physical activity levels as playing the exergames requires the trainee to perform full-body movements to control the game (instead of finger movements on the console).^17^ Prominent examples of the shelve games are Nintendo Wii Sports and XBOX Kinect. Exergame systems used in rehabilitation are Dividat Senso, Sphery ExerCube, or MindMaze. Moreover, meta-analytic evidence suggests a comparable beneficial effect of exergames in rehabilitation on physical and motor functions in comparison to conventional exercising.^17^ Several systematic reviews point towards a superior effect of exergaming for cognitive functions such as executive functions and perception.^17,18^ Exergaming interventions are a relatively new and unconventional training tool to promote physical activity in T2DM^14,19^ and currently, there is a rising body of evidence showing potential beneficial effects of exergaming in diabetic populations with regard to clinical and patient-reported outcomes as well as health behavior patterns. However, the present evidence is limited to heterogeneous intervention types and outcomes studied as well as mostly low study quality. Mostly importantly, there is a highly unmet need for trials focusing on identifying gaming components that contribute to significantly positive effects in addition to sound methodology and reporting.^20^

Specifically, the Dividat Senso is a low-immersive exergame device consisting of a combination of a TV screen and a pressure-sensitive plate containing 20 sensors. These sensors detect timing and position information and transmit it to the screen to provide real-time feedback (visual, auditory, and tactile) enabling the participant to interact with the two-dimensional projection of the video games. The TV screen presents different stimuli, to which the participant must react as fast as possible through whole-body movements like body weight shifting, stepping, walking, squatting, or jumping. Depending on the intervention goal, a wide selection of games is available for targeting different physical and cognitive functions. The exergames were explicitly designed for various psychomotor functions: Focused attention and psychomotor processing speed; target-oriented reaction; divided attention; selective attention and inhibition; static balance; cognitive flexibility; visuo-spatial processing and action planning; working memory and short time memory; visuo-spatial orientation and mental rotation ability; spatial sense; precise step execution action planning.

The Dividat Senso was used in the context of this study as an exergame device with the aim to design a specific exergame-based training protocol. We wanted to test the feasibility in participants with T2DM. The primary endpoint of this study evaluated the feasibility of the specifically designed exergame training protocol in terms of recruitment, adherence, compliance, attrition, satisfaction, acceptance of technology, and motivation under laboratory conditions in T2DM diabetes individuals.^21,22^ We additionally exploratively assessed the possible effects of exergame training on functional outcomes such as daily walking activity, motor-cognitive functions, and HRV as well as patient reported outcomes (PROs). Our research question focused on whether the technology-based exergame program combining cognitive-motor training in T2DM individuals is feasible.

## Materials and methods

### Study design

**Figure 1.**
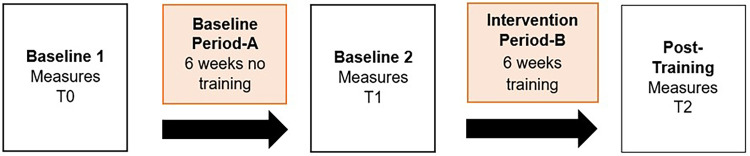
Study procedure including three measurement points with a control and intervention period in between (A-A-B-design).^49^

Participants with T2DM were enrolled in a quasi-experimental single group repeated measures design with a qualitative component for a study period of 12 weeks. This period was divided into two phases, during which they first maintained their daily activity habits for 6 weeks, and then participated in a 6-week cognitive-motor training intervention using a technology-based exergame device. Measurements were taken before starting the first period (baseline T0), right after finishing the first period (baseline T1), and after finishing the second period with the exergame training (post-intervention T2) (see Figure 1). After finalizing the complete study program, a brief interview was conducted to elicit patients’ experiences. The described approach allowed the participants to act in their own control. The study was conducted from April 2021 to March 2022. Each participant was fully informed in writing and orally and signed informed consent according to the latest Declaration of Helsinki prior to any data collection. Ethical approval for the study protocol was obtained from the Research Ethics Committee of ETH Zurich, Switzerland (Protocol No. EK-2021-N-218). All measurements and training were conducted individually one-on-one in the Gait-Lab of the ETH Zurich Hoenggerberg Campus (Zurich, Switzerland). The Consolidated Standards of Reporting Trials (CONSORT) 2010 statement: extension to pilot and feasibility trials and Template for Intervention Description and Replication (TIDieR) were used as the main reference documents and guidelines for transparent trial and intervention reporting, as recommended by Lancaster and Thabane.^23,24^

### Participants

The recommendation for studies such as the present one is a sample size of 12 per group.^25^ The justification for this sample size is based on the rationale of feasibility measures in a new setting with alternative outcomes instead of glucose measurements alone. The recruitment period was from April to December 2021. The recruitment process was conducted in collaboration with the Clinic for Endocrinology, Diabetology and Clinical Nutrition at the University Hospital Zurich (USZ, Zurich, Switzerland). The entire process was supervised by an investigator with more than 20 years of clinical experience in endocrinology and diabetes. Local health professionals (doctors, nurses, diabetes educators and dietitians) screened potential participants and provided initial information about the project using a flyer. In the second step, if interested, the study physician was asked to assess the patient according to the inclusion/exclusion criteria. Participants were included if they had T2DM with glycated hemoglobin (HbA1c) levels between 6.0% and 9.0%, were 50 years of age or older, were able to travel to the study laboratory independently (walking aids allowed), and were physically active only once a week or less in structured training. Potential participants were excluded from the study if they were unable or unwilling to give informed consent, had one (or more) positive items on the PAR-Q without the approval of the study physician, a MoCA score <22,^26,27^ severe musculoskeletal disorders, open plantar ulceration or lower limb amputation, neoplasia, cystic fibrosis, depression or other psychiatric disorders, severe sensory impairment (i.e., vision had to be normal), and were not eligible for the study, i.e., vision must be normal or corrected to normal), type 1 diabetes mellitus, specific diabetes type 3a: maturity onset of the young (MODY), or late autoimmune in diabetes (LADA).

If approved, the patient’s contact details were given to the investigator, who then contacted the potential participant. Other eligible candidates were approached in the waiting room of the clinic and were given verbal and written information. In a final screening step, the German versions of the Physical Activity Readiness Questionnaire (PAR-Q) and the Montreal Cognitive Assessment (MoCA) were administered together with the informed consent. The PAR-Q is a self-screening tool based on the American College of Sports Medicine (ACSM) Standards and Guidelines for Health and Fitness Facilities.^28^ If one or more items were answered ‘yes’, approval had to be obtained from the study physician, otherwise the participant had to be excluded. The MoCA is a widely used screening assessment for cognitive impairment and is an appropriate screening tool in a diabetic population.^29^ The study team approached potential participants and finalized the screening process.

**Figure 2.**
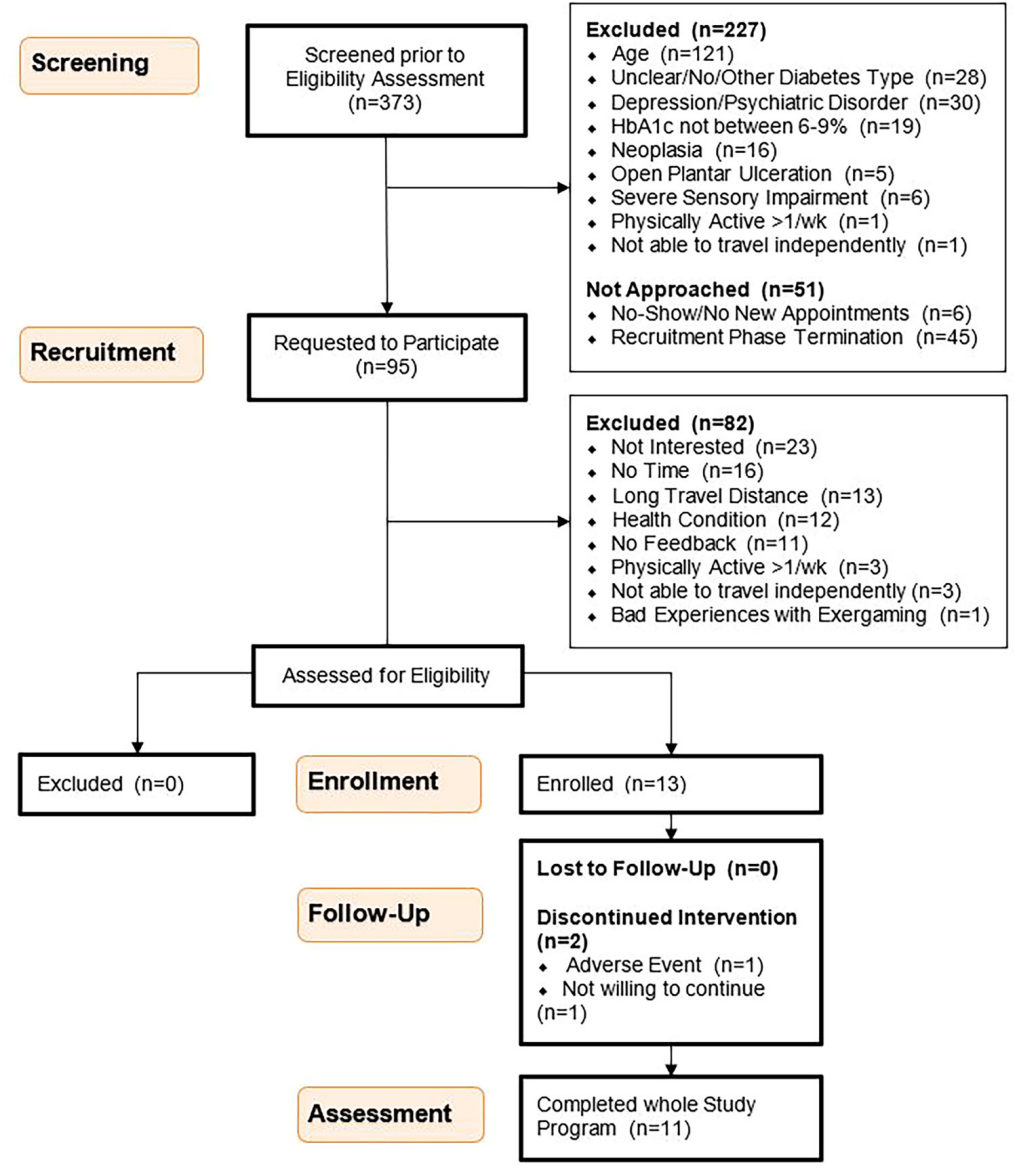
Study flow chart.^50^

Initially, 373 individuals were screened prior to eligibility assessment, of which 278 were directly excluded due to the failure to meet inclusion criteria (*n* = 227) or due to difficulties in approach, such as failure to attend or rescheduling appointments (*n* = 51). Ninety-five potential candidates were approached, resulting in the exclusion of 82 and further eligibility assessment of 13 potential participants. The study flowchart in Figure 2 provides detailed information on the exclusions. All 13 individuals with DM2 (three females and 10 males) passed screening, met all inclusion criteria, signed informed consent and were finally enrolled into the study. One participant dropped out before the second pre-measurement due to the longevity of his problem related to the third adverse event described below. Another participant discontinued participation. This resulted in 11 participants (three women and eight men) completing the entire study.

### Intervention

The frequency, intensity, time, type, volume, and progression (FITT-VP) variables for training prescription were applied for the exergame training planning.^30^ During the six-week period the schedule prescribed two–three trainings per week of moderate-to-high intensity cognitive-motor exergame training. Two sessions per week were mandatory, and the third was on a voluntary basis. As an incentive, to increase adherence and frequency, the participants had the chance to play additional games during the third sessions which were not available during the first two sessions. The game selection targeted different executive functions as planning, divided attention, working memory, and problem solving, which represent functions relevant for people with T2DM.^31^ The duration was planned to be between 23 minute and 30 minutes at the beginning of the program and between 44 and 58 min at the end, including breaks of between one minute and two minutes, respectively. Initially, eight sets of two minutes of active gaming were planned, evolving to 15 sets at the end. Therefore, progression was planned to be manipulated using both, volume and intensity increase. Volume was increased by extending the training duration while simultaneously increasing the number of rounds to play. Intensity alteration is described below. Table 1 shows the specifically designed detailed exergame training plan throughout the complete duration of 6 weeks.

**Table 1.** Intervention exergame training program.

Weeks	Week 1			Week 2			Week 3			Week 4			Week 5			Week 6		
Sessions	1	2	(3)	4	5	(6)	7	8	(9)	10	11	(12)	13	14	(15)	16	17	(18)
Exergames																		
Simple	3	2	3	2	3					1	2		3			2		
Targets	2	3	2	2	2						1	1	2	3	1	3	3	1
Divided	3	3	1	3	2		2	2	1	2		1	2	3	1		3	2
Flexi				2	2	1	3	2	1	2	3	1	2	3	2	3	3	2
Scooper						2	3	3	1	3	3		2	2	1	3	3	
Simon						2	2	3	2	3	3	1	2	2	1	3	3	2
Snake^a^			2			2			2			2			2			2
Habitat^a^						2			2			2			2			2
Hexagon^a^									2			2			2			2
Tetris^a^												2			2			2
Duration (min)	16			18			20		22		24		26		28		30	

*Note*. Exergaming training program sessions per week with number of sets played per game; Every gaming set lasted 2 minutes.

a Bonus-Games possible to play in voluntary sessions marked in parentheses; 2–3 sessions planned per week with 2 “mandatory” sessions and 1 voluntary; Planned breaks 1–2 minutes between every gaming set.

The intensity was assessed at the end of every training using the Borg Category Ratio (CR-10) scale to determine the rate of the perceived exertion (RPE) on a scale from “rest” (rated 0) to “maximum” (rated 10).^32^ Patients were instructed with the Borg rating scale according to the original Borg instructions accompanied by a physical print-out example of the scale. The RPE assessment is a valid method of quantifying subjectively perceived training intensity during diverse types of exercise.^33^

Each single training session was supervised by at least one investigator possessing practical trainee support and guidance experience. For safety reasons, the investigators were trained and supervised by an academic (PhD) physiotherapist with over 20 years of clinical experience in patient care. The exergame device was equipped with a handrail on three sides to provide stability to the trainees when needed.

### Training device

**Figure 3.**
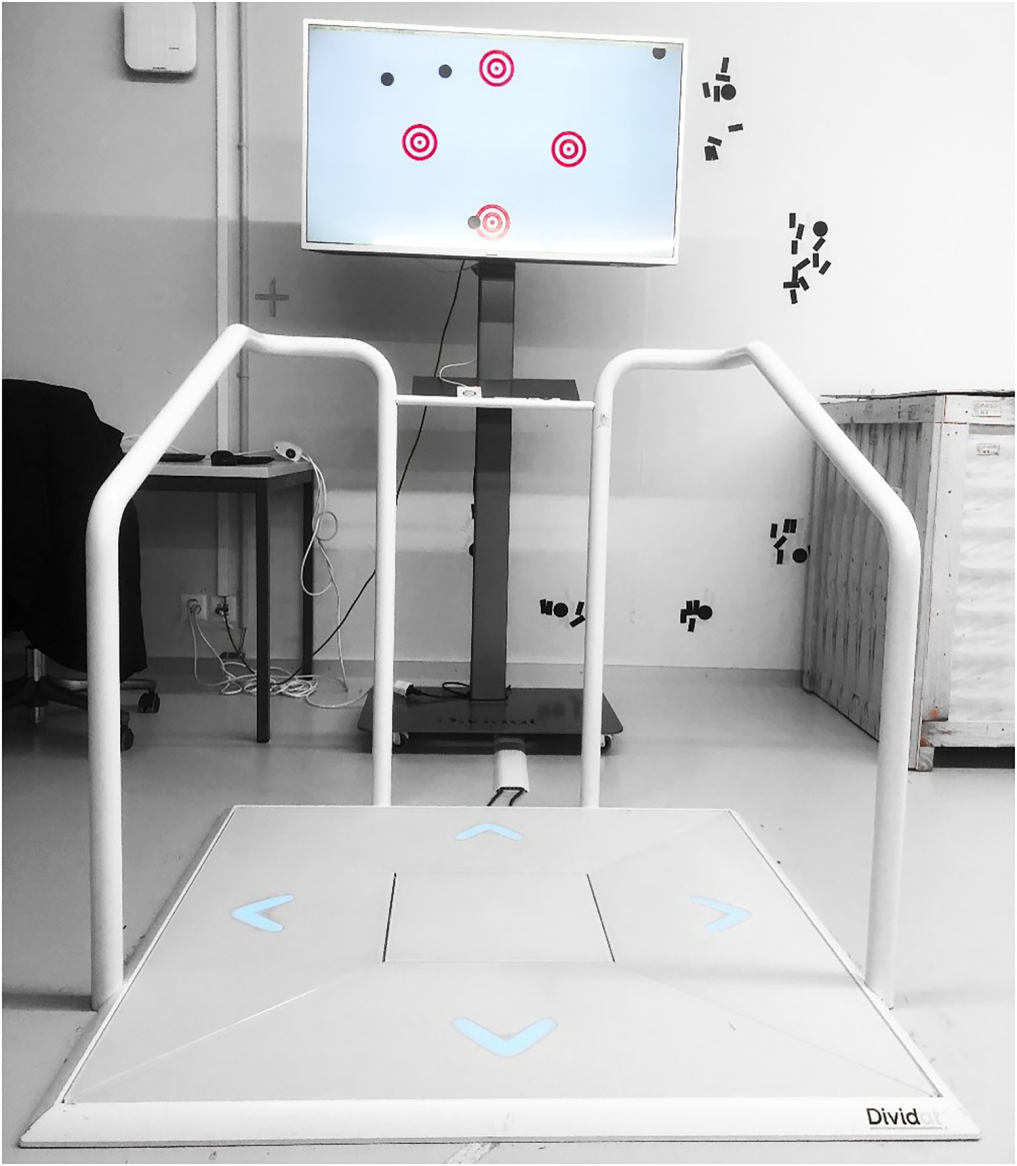
Dividat Senso exergaming device.^49^

The training sessions were performed on the technology-based exergame device Dividat Senso (Dividat AG, Schindellegi, Switzerland; CE Certification) presented in Figure 3. This device was developed for the simultaneous training of physical and cognitive abilities and has been applied in healthy elderly and various patient groups.^34–36^ Pressure-sensitive sensors in the baseplate detect timing and positioning and transmit these to the stimuli presenting a screen to provide real-time feedback (visual, auditory, and tactile), thus enabling the participant to interact with the video game. The training regimen allows the implementation of general training principles like specificity, volume, intensity, variable practice, and progression. Progression and altering intensity are facilitated by game-level adaptations using an intrinsic algorithm that can grade the progression based on the participant's physical and cognitive performance. This allows a simultaneous personalized increase in the complexity and intensity during the gameplay by faster or slower appearing stimuli, ensuring the participant is neither under- nor over-challenged. Table 2 shows an overview of all exergames used in this exergame training protocol, the functions they target, and a short instruction on how to play.

**Table 2.**
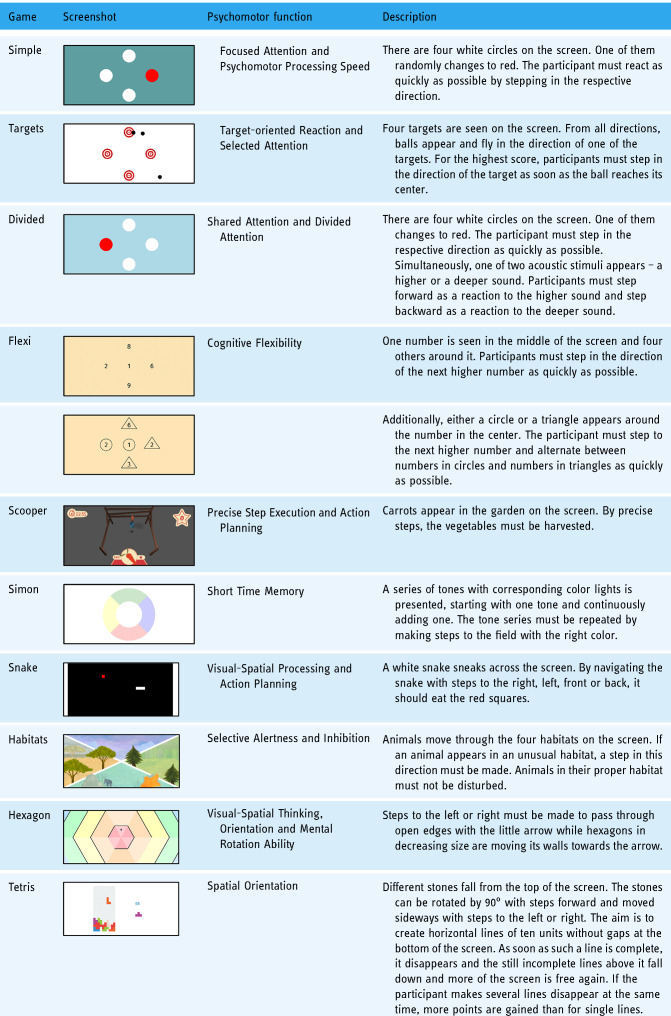
Overview over the Dividat Senso games.

### Primary outcomes

Feasibility outcomes were prioritized in this study, as they were necessary for drawing conclusions that may support modifications of the training approach and planning for further projects, for example, larger-scale randomized controlled studies that assess refined protocols in a home-based setting. Recruitment (enrolled/approached participants), adherence (attended/offered sessions), compliance (mean offered/mean attended training time), and attrition (dropouts/enrolled participants) rates are reported throughout the study.

Furthermore, motivation and satisfaction were assessed using the visual analogue scale (VAS) and the sport and exercise-related self-concordance (SSK) scale.^37–40^ The VAS was assessed after every second training session, whereas the SSK scale was filled out at the measurement time points T1 and T2 (Figure 1). The VAS uses one question for each factor and a 10 cm long bar with anchor descriptors.^38^ Assessing satisfaction in individuals with T2DM this way is more reliable than using classic Likert scales and less vulnerable to bias from confounding factors.^37,38^ The SSK-scale relies on given options.^39^ Self-concordance, also called “ego-distance,” is described as the extent to which a goal reflects personal interests and values and can help measure the ego-distance of an exercise-specific intention. The SSK-Scale is both conceptually and methodologically a robust instrument to measure exercise-related self-concordance.^40^

During post-intervention measurements, technology acceptance was assessed using the technology acceptance model (TAM) questionnaire. The model assesses a users’ behavioral intentions towards the usage of new technologies. Using a 7-point Likert scale ranging from “strongly disagree” (rated 1) and “strongly agree” (rated 7), the intention to use a system is determined by the perceived usefulness (PU), perceived ease of use (PEOU), attitude towards using (ATU), and behavioral intention to use (BITU).^41,42^

For the evaluation of all the feasibility parameters an *a priori* determined protocol was using thresholds established from comparable studies: Recruitment rate should be ≥20%, adherence rate ≥70%, compliance rate ≥70%, attrition rate ≤20%, mean motivation ≥6 (scale 0–10) or 60%, mean satisfaction ≥60%, and technology acceptance subscales ≥4.^43–46^ Depending on the outcomes with respect to these thresholds, consequences were defined determining the further measures that must be taken to modify the intervention approach (see Table 3).

**Table 3.** Conclusions and consequences drawn based on the feasibility results.

Result	Consequences
All feasibility criteria are met	Intervention is feasible, the RCT can be conducted without modifications.
Recruitment rate is too low	Evaluate the reasons for failed recruitments: In case of intervention-unrelated reasons only, reconsider the recruitment procedure, but conduct the intervention without modifications in the RCT. In case of intervention-related reasons, implement appropriate modifications to the intervention for the RCT.
Attrition rate is too high	Evaluate reasons for dropouts: In case of training-unrelated dropouts only, continue with RCT without modifications. In case of training-related dropouts, modifications must be undertaken to hinder similar events in the RCT. In case of a drop-out due to a training-related serious adverse event, the RCT must not be conducted.
Adherence, compliance, motivation, satisfaction rates are too low	Evaluate the reasons for the unsatisfactory results and implement according to minor modifications for the RCT.
≥4/7 feasibility criteria are not met	Evaluate the reasons and implement major modifications for the RCT.
≥6/7 feasibility criteria are not met	RCT shall be reconsidered.

*Note*. RCT = randomized controlled trial.

### Secondary outcomes

A standardized measurement procedure was used at every measurement time point (T0, T1, T2). First, participants filled in all questionnaires. This also allowed them to get acquainted and at ease with the investigation environment. Thereafter, HRV was assessed, followed by the gait analysis. In the end, the cognitive-motor tests were conducted.

HRV, as a surrogate measure of the interaction between the brain and the cardiovascular system,^47^ was measured at T0, T1 and T2. A one-lead electrocardiogram (ECG) was performed recording beat-to-beat R-R intervals using a wearable textile chest belt with integrated electrodes (Unico Swiss Tex GmbH, Alpnach, Switzerland). Data collection, storage, readout, and reporting was performed by using a Faros logger (Bittium Biosignals Oy, Kuopio, Finland) as well as Kubios’ HRV Standard analysis software (version 3.5.0, Kubios Oy, Kuopio, Finland) by following the methodology of Eggenberger et al.^6,48–51^

Gait function was assessed using the Physilog®5 (Gait Up SA, Renens, Switzerland) wearable sensors for measurement and the companies’ Gait Up software for the analysis. This gait analysis tool kit provides a quantitative, objective, and valid assessment of gait movement. Spatial (distance) and temporal (time) gait parameters, including speed, variability, and finally mean and standard deviation of cycle duration, cadence and stride length, stride velocity, stance, swing, double support, swing width, and minimum toe clearance were measured. The wearable standalone inertial movement sensors (50 × 37 × 9.2 mm, 19 g, anatomical curved shape) were attached to the back of the feet via an elastic strap using a Velcro fastener for fixation. The Functional Gait Assessment (FGA) was used to standardize the assessment procedure.^52^ Prior to testing, participants received standardized instruction and a visual demonstration. Thereafter, the participants were allowed to try the procedure once, finally followed by the real test. Four different tasks out of the FGA were chosen for the gait assessments, whereby for each task the defined walking path had to be completed three times in a row with turns in a self-comfortable manner: (a) Gait Level Surface—Normal Speed; (b) Gait Level Surface—Fastest Speed; (c) Gait with Narrow Base of Support; (d) Gait and Dual Task. This allowed the assessment of sufficient steps for assessing variability measures.^53^ During dual tasking, the participants had to solve a simple mathematical task while walking. This consisted of walking with their preferred walking speed, while continuously counting backwards in steps of seven starting from 100. To eliminate learning effects and bias the participants conducted the same task, however, in steps of nine starting from 100 during trying out. In addition to spatio-temporal gait parameters, the relative dual-task cost (DTC) of walking, as the percentage of loss relative to the single-task walking performance was calculated using the following formula:^54^

$$\mathrm{DTC}\;[\text{\%}]=100\frac{single\;task\;score-dual\;taskscore}{single\;task\;score}$$

To analyze steady-state walking, acceleration and deceleration steps as well as turns were removed by the software. Measurements were taken at T0, T1, and T2.

For the detection of changes in daily physical activity patterns pre- and post-intervention the StepWatch (Modus Health LLC, Edmonds, WA, USA) accelerometers and corresponding StepWatch software (version 3.4) were used. The device is a valid and reliable tool to determine the number of strides per day.^55^ Participants were instructed to wear the StepWatch on the ankle of the dominant leg for 7 consecutive days within the first and the last week of the exergame training. Before handing it out, the device had to be individually calibrated for every single participant. As part of the calibration process, initially, a few questions had to be answered. The StepWatch was calibrated by walking 40 steps, while the investigator counted the steps. The participants were encouraged to store the device on the bedside table and to put it on immediately after waking up. Intensity, variability, and pattern of activity were derived and further analyzed with the activity at different intensity levels throughout the day.^56^ Norm values, recommendations, and comparative values for physical activity levels are available for healthy participants and chronic disease populations.^57^

All cognitive-motor assessments were conducted on the exergame device Dividat Senso. The tests were performed at T0, T1, and T2 in the following order: Stroop Test, GoNogo Test, Reaction Time Test, and Flexibility Test. First, a short practice session was provided for familiarization. Aggregated reaction times in ms and error ratios were defined as outcome variables. Table 4 shows a detailed overview of the assessments, the assessed function, and a concise description of the task.

**Table 4.**
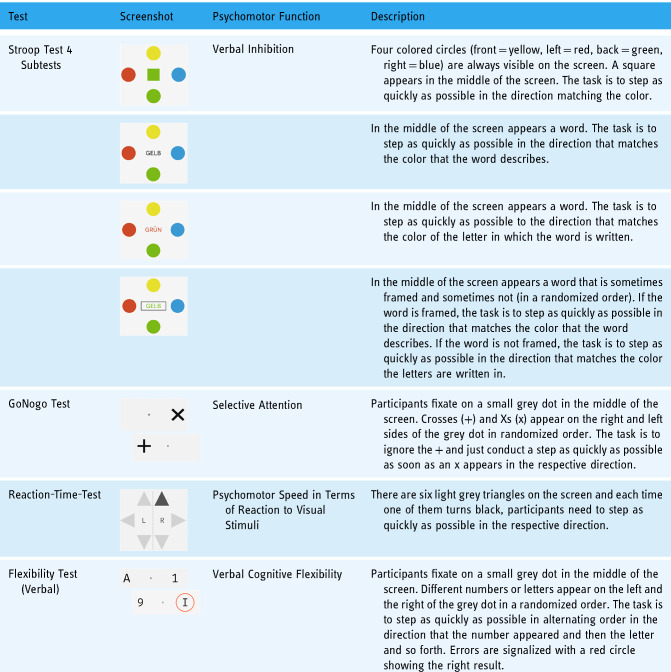
Overview over the Dividat Senso cognitive assessments.

As a measurement tool for PROs, the Appraisal of Diabetes Scale (ADS) was provided as a paper-form questionnaire. This brief 7-item scale is a health-related quality of life measure (HR-QoL) for individuals with diabetes and measures their appraisal of diabetes on a 5-point Likert scale from “total disagreement” (1) to “total agreement” (5). Every answer can be counted as a score and with a simple two-step calculation this qualitative measure can be converted into a quantitative outcome. The scale has been shown to be a valid measure for diabetes-related appraisal.^58^

Patient-related experience measures (PREMs) using the Dividat Senso exergames were explored through individual semi-structured interviews after the study program completion. A guideline with predetermined but open-ended questions was employed. The interviews were audiotaped and transcribed verbatim.

### Statistical analysis

Following a per-protocol analysis, only the results of the participants that adhered to the training protocol and did not drop out were counted. All statistical analyses and calculations were performed using RStudio and R version 4.1.2 software package (RStudio, Inc., Boston, MA, United States). Clinical and feasibility data were calculated using descriptive statistics including average percentages of the corresponding values and calculated overall ranges. Secondary outcomes (HRV, gait analysis, cognitive-motor tests, step activity) were tested for normality using the Shapiro–Wilk test. Depending on the evaluation, a one-way repeated measures analysis of variances (ANOVA) or the Friedman Test was used for the comparison of the normally or non-normally distributed results. If significant, Bonferroni-corrections for ANOVA, and Dunn-Bonferroni for Friedman Tests, were carried out as *post hoc* analyses. For pre vs. post comparisons, a paired *T*-Test or the Wilcoxon Test was carried out for normally and non-normally distributed data, respectively. No imputation strategies were followed or carried out for missing values and a complete case analysis was performed in this case. Additionally, mean and standard deviation (SD) were reported for interval data and median and inter-quartile range for ordinal data. The level of statistical significance was defined as *p *< .05, whereas *p* < .1 was reported as a trend towards significance. Finally, effect estimates were analyzed for within-group differences including 95% confidence intervals (CI) for estimation interpretation.^59–61^ For non-normally distributed data, effect sizes were expressed as *r* = z/√N, where z describes the approximation of the observed difference in terms of the standard normal distribution and *N* is the variable for the total number of samples (*r* = 0.1, small effect; *r* = 0.3, medium effect; and *r *= 0.5, large effect).^62^ Cohen's $d=\frac{MA-MB}{\sigma }$ was reported, where MA and MB describe the mean of timepoint A and B, respectively, and σ is the standard deviation (*d* 0.2, small effect, *d* 0.5, medium effect, *d* 0.8, large effect) for normally distributed data.^63^

### Qualitative analysis

The anonymized transcripts were entered into NVivo Version 20 (Alfasoft GmbH, Frankfurt am Main, Germany) for data management and analysis. Data were analyzed using qualitative content analysis.^64^ After reading each transcript, sentences and paragraphs were coded inductively and preliminary categories were developed. This process was continued until final categories were identified. The selected quotes were cleaned up for filler words and repetitions for better readability without changing their meaning and then translated into English. The translation of the codes was validated by a native speaker. Data analysis was performed by one member of the research team but discussed regularly with the principal investigator (PI) who was present as an observer for several of the interviews. There was general agreement that the results reflected the participants’ experiences. As we interviewed all participants, we aimed for data saturation on the level of the individual interview. Probing continued until saturation on the individual level was achieved.^65^

## Results

### Primary outcomes

The recruitment rate was 13.7% in nine months, whereas the attrition rate with two drop-outs resulted in 15.4%. Mean age was 60.4 (*SD*: 7.2, range: 50–74) years. Table 5 gives insight into the exact demographic and clinical data of the analyzed sample. Both recruitment and attrition rates yielded values lower than the predefined thresholds of ≥20% and ≤20%, respectively, implying insufficiencies in the recruitment process. In total, five non-intervention-related adverse events (AE) occurred, including pain accompanied by overall weakness and headaches, bruises and abrasions, having a cold, and a small surgery not leading to hospitalization but to weakness and pain when moving. No serious adverse events were reported.

**Table 5.** Clinical and demographic characteristics of the analyzed sample (*n* = 11).

Participant characteristics	Mean	SD	Median	Range
Age (years)	60.4	7.2	59	50–74
Height (cm)	168	0.1	169	150–181
Weight (kg)	87.7	20.7	83	54–127
BMI (kg/m^2^)	30.9	5.6	32.2	24–41.5
Waist circumference (cm)	105.5	14.4	102	86–130
Years of disease (years)	8	8.7	4	0–24
HbA1c (%)	7.4	1.1	6.9	6.0–8.8
Charlson Comorbidity Index	3	0.9	3	2–4
Systolic blood pressure (mmHg)	137	19.1	135	101–169
Diastolic blood pressure (mmHg)	84	16.7	82	52–114
MoCA	24	2.6	24	22–30
Sex	*n*	*%*		
Female	3	27		
Male	8	73		
Highest education level	*n*	*%*		
High school	3	27		
Apprenticeship	4	36.5		
University	4	36.5		

*Note*. SD = standard deviation.

**Figure 4.**
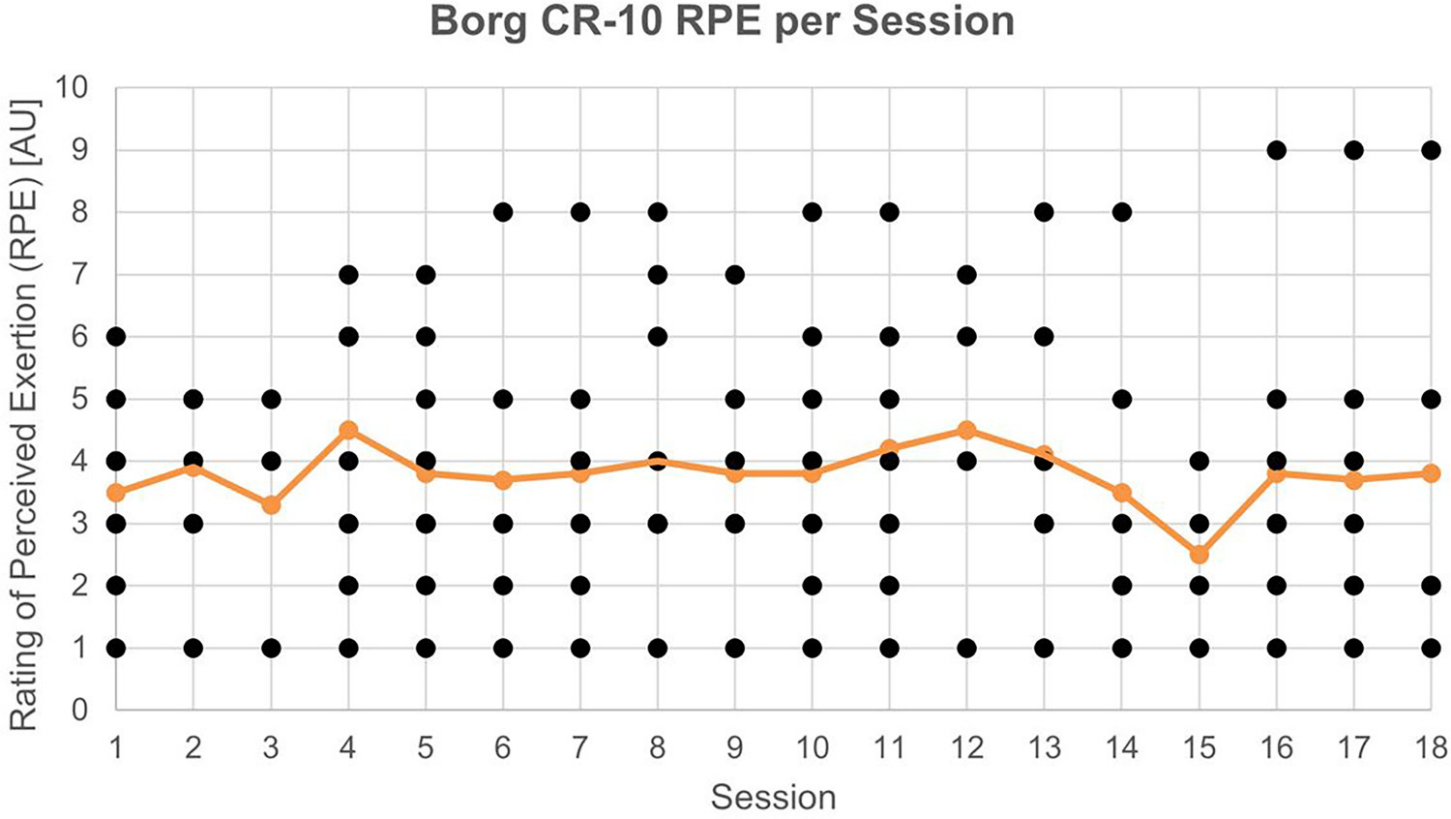
Individual (dark dots) and group mean (light dots combined with line) session rating of perceived exertion (RPE) across the 18 possible exergaming sessions. AU = arbitrary unit.^51^

Training sessions lasted on average between 23.8 (*SD*: 1.8, range: 20.8–26.2) minutes at the beginning and 45 (*SD*: 3.5, range: 41.7–52.1) minutes at the end of the intervention. Overall mean training duration was 31.8 (*SD*: 7.7, range: 20.8–52.5) minutes per session with a mean frequency of 2.1 (*SD*: 0.30, range: 1.82–2.55) sessions per week. Referring to the two necessary weekly sessions, the adherence rate was 86.4% (attendance rate: 114/132 sessions). Taking into consideration the third voluntary session, adherence resulted in 70.2% (attendance rate: 139/198 sessions). The compliance rate was 99.7% (minutes played: 3052/3060 offered play minutes from attended sessions). All three rates lay above the ≥70% cutoff values, initially regarded as acceptable. Mean intensity was 3.9 (*SD*: 2.0, range: 1–9) over all sessions corresponding to between “moderate” (Borg CR-10: 3) and “somewhat strong” (Borg CR-10: 4) in verbal equivalents, lying in the predefined target intensity of moderate-to-high. Figure 4 shows the individual and group mean session ratings in detail.

Table 6 provides insight into the VAS and the TAM outcome variables. VAS results reflect high motivation and satisfaction, both lying over the threshold values of ≥6. TAM reveals an overall remarkably high mean ATU and high mean values for PU, PEOU, and BITU. In general, all subscales provided values above the predefined cutoff of ≥4 and satisfactorily technology acceptance. Regarding the motivation values measured by the SSK-Scale, the T1 measurement showed a mean SSK-Index of 13.1 (*SD*: 12.2, median: 7, range: 0–29) compared to 15.6 (*SD*: 7.0, median: 14, range: 5–30) in T2. Data was normally distributed, and no significant difference was revealed by paired two-sided *T*-Test for the SSK indices [*T*(10) = 1.036, *p *= .325]. The corresponding effect size was small (*d *= 0.31).

**Table 6.** Evaluation of the visual analog scale and the technology acceptance model.

Visual Analog Scale	Mean	SD	Median	Range
Motivation	8.2	1.3	8.3	6.5–9.9
Satisfaction	8.0	1.3	8.4	6.0–9.7
Technology Acceptance Model				
Perceived Ease of Use (PEOU)	5.8	1.2	6.0	3–7
Perceived Usefulness (PU)	6.3	0.9	6.8	5–7
Attitude Toward Using (ATU)	6.7	0.6	7.0	5–7
Behavioral Intention to use (BITU)	6.2	0.9	6.3	4–7

*Note*. SD = standard deviation.

### Secondary outcomes

HRV data were normally distributed for the indices’ standard deviation of the normal-to-normal intervals (SDNN), absolute power of low-frequency band (LF), and Poincaré plot standard deviation along the line of identity (SD2) only. One-way multiple measures ANOVA with sphericity assumed and the non-parametrical equivalent Friedman Test revealed no statistically significant differences between time-points for any of the HRV indices. The effect sizes were small for all parameters with the exception of the LF/HF ratio, which had a medium effect size (*r* = .37). Table 7 shows an in-depth view of the results.

**Table 7.** Overview heart rate variability outcomes.

HRV Indices	Mean (SD)					
ANOVA	Pre-T0	Pre-T1	Post-T2	*F*(2, 20)	*p*	*d*
SDNN (ms)	2.82 × 10^−5^ (9.13 × 10^−6^)	2.81 × 10^−5^ (8.43 × 10^−6^)	2.87 × 10^−5^ (1.24 × 10^−5^)	0.008	.992	0.03
95% CI	[2.28 × 10^−5^, 3.36 × 10^−5^]	[2.32 × 10^−5^, 3.31 × 10^−5^]	[2.14 × 10^−5^, 3.6 × 10^−5^]			
LF (ms^2^)	1.90 × 10^−4^ (1.69 × 10^−4^)	1.46 × 10^−4^ (9.74 × 10^−5^)	1.26 × 10^−4^ (9.64 × 10^−5^)	1.256	.306	0.17
95% CI	[9.01 × 10^−5^, 2.9 × 10^−4^]	[8.83 × 10^−5^, 2.03 × 10^−4^]	[6.87 × 10^−5^, 1.83 × 10^−4^]			
SD2 (ms)	18.83 (6.44)	18.23 (6.60)	16.44 (5.35)	0.679	.518	0.21
95% CI	[15.03, 22.63]	[14.34, 22.13]	[13.28, 19.61]			

Friedman Test	Pre-T0	Pre-T1	Post-T2	χ^2^(2)	*p*	*r*
RMSSD (ms)	3.71 × 10^−8^ (1.37 × 10^−8^)	4.06 × 10^−8^ (1.36 × 10^−8^)	4.95 × 10^−8^ (4.35 × 10^−8^)	0.727	.695	0.13
95% CI	[2.9 × 10^−8^, 4.51 × 10^−8^]	[3.26 × 10^−8^, 4.87 × 10^−8^]	[2.38 × 10^−8^, 7.52 × 10^−8^]			
PNN50 (%)	1.22 × 10^−20^ (2.35 × 10^−20^)	1.10 × 10^−20^ (1.58 × 10^−20^)	4.79 × 10^−20^ (1.25 × 10^−19^)	1.027	.598	0.20
95% CI	[−1.7 × 10^−21^, 2.61 × 10^−20^]	[1.7 × 10^−21^, 2.03 × 10^−20^]	[−2.6 × 10^−20^, 1.22 × 10^−19^]			
HF (ms^2^)	2.74 × 10^−10^ (2.42 × 10^−10^)	3.15 × 10^−10^ (2.70 × 10^−10^)	2.54 × 10^−10^ (2.30 × 10^−10^)	0.727	.695	0.16
95% CI	[1.3 × 10^−10^, 4.17 × 10^−10^]	[1.6 × 10^−10^, 4.74 × 10^−10^]	[1.18 × 10^−10^, 3.9 × 10^−10^]			
LF/HF (%)	4.31 × 10^−6^ (4.78 × 10^−6^)	2.47 × 10^−6^ (1.80 × 10^−6^)	3.06 × 10^−6^ (2.48 × 10^−6^)	1.273	.529	0.37
95% CI	[1.48 × 10^−6^, 7.13 × 10^−6^]	[1.41 × 10^−6^, 3.54 × 10^−6^]	[1.6 × 10^−6^, 4.53 × 10^−6^]			
SD1 (ms)	13.33 (9.84)	13.53 (10.84)	11.42 (5.64)	0.182	.913	0.19
95% CI	[7.52, 19.15]	[7.13, 19.94]	[8.09, 14.75]			

*Note*. HRV = heart rate variability; SD = standard deviation; ANOVA = analysis of variances; *F*(2,20) = test statistic of ANOVA (degrees of freedom); χ^2^(2) = test statistic Friedman Test (degrees of freedom); CI = confidence interval; SDNN = standard deviation of the normal-to-normal intervals; LF = absolute power of low-frequency band; SD2 = poincaré plot standard deviation along the line of identity; RMSSD = root mean square of successive R-R interval differences; PNN50 percentage of adjacent normal-to-normal intervals that differ by more than 50 ms; HF = absolute power of high-frequency band; LF/HF = ratio of LF to HF power; SD1 = poincaré plot standard deviation perpendicular to the line of identity; *p* = *p*-value all timepoints (level of significance); Effect sizes between T1 vs. T2: Cohen's d for parametric and r for non-parametric data.

Table 8 gives a detailed overview of all gait analysis outcomes. Except for variability, cycle duration, cadence, and swing width, normal speed condition testing data were normally distributed. ANOVA detected a significant difference between time-points for stride velocity [*F*(2, 20) = 6.843, *p *= .005*]. *Post hoc* Bonferroni correction revealed a significant positive change (*p *= .005*) between timepoints T0 and T1 [1.03 (*SD*: 0.23, 95% CI: 0.89; 1.16) ms^−1^ vs. 1.13 (*SD*: 0.19, 95% CI: 1.02; 1.24) ms^−1^, respectively] with a large effect size (*d*: 1.31). The Friedman Test found a significant difference between time-points for cycle duration [χ^2^(2) = 8.909, *p *= .012*]. The effect size between T1 and T2 was medium-to-large (*r* = .48). Dunn-Bonferroni-corrected *post hoc* tests revealed a significant decrease [z = −2.67, *p *= .008*] in the cycle duration between timepoints T0 and T1 [1.20 (*SD*: 0.08, 95% CI: 1.15; 1.24) s versus 1.12 (*SD*: 0.10, 95% CI: 1.06; 1.18) s, respectively] with a large effect size (*r* = .70). No significant differences were shown for the other gait parameters and the corresponding intervention effect sizes were small. Furthermore, no significant differences were shown between timepoints T1 and T2.

**Table 8.** Overview gait analysis outcomes.

Gait indices		Mean (*SD*)					Effect size
	*n*	Pre-T0	Pre-T1	Post-T2	F/χ^2^	*p*	
Variability (%)							
Normal speed	11	13.72 (17.33)	6.91 (5.59)	10.35 (12.61)	5.636^b^	.060**	0.03^e^
95% CI		[3.49; 23.96]	[3.61; 10.21]	[2.89; 17.80]			
Fastest speed	11	13.96 (12.26)	12.10 (13.20)	12.07 (11.16)	2.364^b^	.307	0.11^e^
95% CI		[6.72; 20.68]	[4.30; 16.39]	[5.47; 17.54]			
Narrow BoS	11	39.78 (44.67)	20.89 (13.08)	31.59 (17.51)	5.636^b^	.060**	0.59^e^
95% CI		[13.39; 66.18]	[13.16; 28.62]	[21.25; 41.94]			
Dual task	11	41.76 (34.50)	37.44 (21.17)	35.45 (21.58)	0.199^a^	.821	0.06^e^
95% CI		[21.37; 62.15]	[24.93; 49.95]	[22.70; 48.20]			
DTC (%)	11	510.63 (697.52)	595.24 (423.05)	573.85 (594.21)	2.364^b^	.307	0.08^e^
95% CI		[98.43; 922.83]	[345.23; 845.24]	[222.70; 925.00]			
Cycle duration (s)							
Normal speed	11	1.20 (0.08)	1.12 (0.10)	1.15 (0.07)	8.909^b^	.012*	0.70^d^
95% CI		[1.15; 1.24]	[1.06; 1.18]	[1.11; 1.19]		.008*^,c,d^	0.48^e^
Fastest speed	11	1.04 (0.22)	1.00 (0.15)	0.98 (0.11)	1.636^b^	.441	0.19^e^
95% CI		[0.91; 1.96]	[0.91; 1.91]	[0.92; 1.90]			
Narrow BoS	11	2.33 (0.71)	2.09 (0.66)	2.04 (0.44)	4.447^a^	.025*	0.16^e^
95% CI		[1.91; 2.75]	[1.70; 2.48]	[1.77; 2.30]		.093**^,c,f^	
Dual task	11	2.85 (1.62)	2.15 (0.93)	2.26 (0.99)	0.727^b^	.695	0.05^e^
95% CI		[1.89; 3.81]	[1.60; 2.69]	[1.67; 2.85]			
DTC (%)	11	137.11 (133.20)	92.54 (90.01)	100.65 (98.05)	0.727^b^	.695	0.27^e^
95% CI		[58.40; 215.83]	[39.35; 145.74]	[42.70; 158.59]			
Cadence (steps/min)							
Normal speed	11	102.28 (5.63)	108.10 (8.00)	106.57 (5.77)	5.636^b^	.060**	0.43^e^
95% CI		[98.96; 105.61]	[103.37; 112.83]	[103.16; 109.99]			
Fastest speed	11	120.95 (16.96)	124.49 (15.84)	125.35 (12.54)	0.786^a^	.469	0.12^e^
95% CI		[110.93; 231.88]	[115.13; 239.63]	[117.94; 243.29]			
Narrow BoS	11	62.87 (19.40)	65.47 (18.62)	67.07 (13.72)	1.463^a^	.255	0.19^e^
95% CI		[51.40; 74.33]	[54.46; 76.47]	[58.97; 75.18]			
Dual task	11	59.57 (20.63)	67.65 (16.64)	66.62 (18.97)	1.757^a^	.198	0.07^e^
95% CI		[47.38; 71.77]	[57.82; 77.49]	[55.41; 77.83]			
DTC (%)	11	41.85 (19.85)	37.24 (15.12)	36.95 (19.38)	0.735^a^	.492	0.02^e^
95% CI		[30.12; 53.58]	[28.30; 46.18]	[25.50; 48.41]			

*Note*. SD = standard deviation; *n* = sample size included in test; CI = confidence interval; BoS = base of support; DTC = dual task cost.

a ANOVA, *F*(2, 20), Cohen's *d*.

b Friedman test.

c Post hoc test.

d Indicating comparison between T0 and T1.

e Indicating comparison between T1 and T2.

f Indicating comparison between T0 and T2, χ^2^(2) *r*; *p*-value all timepoints.

* *p* < .05; **p < .1, trend.

**Table 8.** Continued (1).

Gait indices		Mean (*SD*)					Effect size
	*n*	Pre-T0	Pre-T1	Post-T2	F/χ^2^	*p*	
Stride length (m)							
Normal speed	11	1.18 (0.23)	1.24 (0.19)	1.23 (0.19)	2.131^a^	.145	0.15^e^
95% CI		[1.05; 1.32]	[1.13; 1.35]	[1.12; 1.34]			
Fastest speed	11	1.37 (0.26)	1.42 (0.21)	1.45 (0.18)	1.963^a^	.167	0.49^e^
95% CI		[1.21; 2.58]	[1.30; 2.72]	[1.34; 2.79]			
Narrow BoS	11	0.54 (0.15)	0.53 (0.10)	0.53 (0.09)	0.125^a^	.883	0.12^e^
95% CI		[0.45; 0.63]	[0.47; 0.59]	[0.47; 0.58]			
Dual task	11	0.96 (0.32)	1.01 (0.29)	1.00 (0.30)	0.348^a^	.710	0.11^e^
95% CI		[0.77; 1.15]	[0.84; 1.18]	[0.82; 1.18]			
DTC (%)	11	19.38 (22.46)	19.27 (17.95)	20.23 (19.88)	0.545^b^	.761	0.03^e^
95% CI		[6.10; 32.65]	[8.67; 29.88]	[8.48; 31.98]			
Stride velocity (m/s)							
Normal speed	11	1.03 (0.23)	1.13 (0.19)	1.10 (0.18)	6.843^a^	.005*	1.31^d^
95% CI		[0.89; 1.16]	[1.02; 1.24]	[1.00; 1.21]		.005*^,c,d^	0.31^e^
Fastest speed	11	1.43 (0.31)	1.50 (0.24)	1.54 (0.24)	2.182^b^	.336	0.62^e^
95% CI		[1.25; 2.67]	[1.36; 2.86]	[1.40; 2.94]			
Narrow BoS	11	0.34 (0.15)	0.37 (0.15)	0.36 (0.12)	0.967^a^	.397	0.05^e^
95% CI		[0.26; 0.43]	[0.28; 0.45]	[0.29; 0.44]			
Dual task	11	0.53 (0.31)	0.62 (0.26)	0.59 (0.27)	1.649^a^	.217	0.18^e^
95% CI		[0.34; 0.71]	[0.46; 0.77]	[0.44; 0.75]			
DTC (%)	11	50.78 (24.19)	46.96 (17.77)	46.70 (22.83)	0.351^a^	.708	0.02^e^
95% CI		[36.49; 65.08]	[36.46; 57.46]	[33.21; 60.19]			
Stance (% of cycle duration)							
Normal speed	11	62.98 (3.58)	61.97 (2.48)	62.50 (2.65)	1.517^a^	.243	0.36^e^
95% CI		[60.86; 65.09]	[60.51; 63.44]	[60.94; 64.07]			
Fastest speed	11	60.87 (3.25)	60.79 (2.71)	60.06 (2.33)	2.364^b^	.307	0.59^e^
95% CI		[58.95; 119.81]	[59.18; 119.97]	[58.68; 118.73]			
Narrow BoS	11	73.72 (5.12)	72.70 (5.10)	71.94 (3.77)	1.920^a^	.173	0.22^e^
95% CI		[70.70; 76.75]	[69.69; 75.72]	[69.72; 74.17]			
Dual task	11	69.61 (9.35)	67.48 (7.88)	68.02 (6.91)	0.182^b^	.913	0.11^e^
95% CI		[64.09; 75.14]	[62.83; 72.14]	[63.93; 72.10]			
DTC (%)	11	10.57 (13.70)	8.72 (9.85)	8.84 (10.20)	2.909^b^	.234	0.13^e^
95% CI		[2.47; 18.66]	[2.89; 14.54]	[2.81; 14.87]			

**Table 8.** Continued (2).

Gait indices			Mean (*SD*)				Effect Size
	*n*	Pre-T0	Pre-T1	Post-T2	F/χ^2^	*p*	
Swing (% of cycle duration)							
Normal speed	11	37.02 (3.58)	38.03 (2.48)	37.50 (2.65)	1.517^a^	.243	0.36^e^
95% CI		[34.91; 39.14]	[36.56; 39.49]	[35.93; 39.06]			
Fastest speed	11	39.13 (3.25)	39.21 (2.71)	39.94 (2.33)	2.364^b^	.307	0.59^e^
95% CI		[37.22; 76.35]	[37.61; 76.82]	[38.57; 78.51]			
Narrow BoS	11	26.28 (5.12)	27.30 (5.10)	28.06 (3.77)	1.920^a^	.173	0.22^e^
95% CI		[23.25; 29.30]	[24.28; 30.31]	[25.83; 30.28]			
Dual task	11	30.39 (9.35)	32.52 (7.88)	31.98 (6.91)	0.182^b^	.913	0.11^e^
95% CI		[24.86; 35.91]	[27.86; 37.17]	[27.90; 36.07]			
DTC (%)	11	18.20 (23.42)	15.05 (17.93)	14.80 (17.16)	1.636^b^	.441	0.13^e^
95% CI		[4.36; 32.04]	[4.46; 25.65]	[4.66; 24.94]			
Double support (% of cycle duration)							
Normal speed	11	25.08 (6.93)	23.00 (5.13)	23.78 (4.21)	1.827^a^	.187	0.29^e^
95% CI		[20.99; 29.18]	[19.97; 26.03]	[21.29; 26.27]			
Fastest speed	11	20.90 (7.59)	19.26 (4.45)	18.30 (4.86)	0.727^b^	.695	0.35^e^
95% CI		[16.41; 37.31]	[16.63; 35.90]	[15.43; 33.73]			
Narrow BoS	11	49.29 (13.23)	46.66 (11.50)	44.21 (9.55)	2.893^a^	.079^‡^	0.29^e^
95% CI		[41.47; 57.11]	[39.87; 53.46]	[38.57; 49.86]			
Dual task	11	37.24 (21.01)	31.24 (15.52)	32.42 (17.07)	2.909^b^	.234	0.30^e^
95% CI		[24.82; 49.66]	[22.06; 40.41]	[22.33; 42.51]			
DTC (%)	11	50.15 (77.19)	32.17 (44.56)	33.43 (57.47)	0.727^b^	.695	0^e^
95% CI		[4.53; 95.77]	[5.84; 58.50]	[−0.53; 67.39]			
Swing width (m)							
Normal speed	11	0.05 (0.01)	0.05 (0.02)	0.05 (0.02)	2.182^b^	.336	0.27^e^
95% CI		[0.04; 0.06]	[0.05; 0.06]	[0.04; 0.06]			
Fastest speed	11	0.05 (0.01)	0.05 (0.02)	0.06 (0.01)	0.182^b^	.913	0.16^e^
95% CI		[0.05; 0.10]	[0.04; 0.10]	[0.05; 0.10]			
Narrow BoS	11	0.09 (0.02)	0.09 (0.02)	0.10 (0.02)	0.225^a^	.801	0.15^e^
95% CI		[0.08; 0.10]	[0.08; 0.11]	[0.08; 0.11]			
Dual task	11	0.05 (0.03)	0.06 (0.02)	0.06 (0.03)	0.049^a^	.952	0.06^e^
95% CI		[0.03; 0.07]	[0.04; 0.07]	[0.04; 0.07]			
DTC (%)	11	15.81 (85.62)	5.40 (46.63)	11.05 (54.37)	0.182^b^	.913	0.11^e^
95% CI		[−34.79; 66.40]	[−22.16; 32.96]	[−21.08; 43.18]			

**Table 8.** Continued (3).

Gait indices		Mean (*SD*)					Effect size
	*n*	Pre-T0	Pre-T1	Post-T2	F/χ^2^	*p*	
Minimal toe clearance (m)							
Normal speed	11	0.02 (0.01)	0.02 (0.01)	0.02 (0.01)	0.829^a^	.451	0.37^e^
95% CI		[0.02; 0.03]	[0.01; 0.03]	[0.02; 0.03]			
Fastest speed	11	0.02 (0.01)	0.02 (0.01)	0.02 (0.01)	0.747^a^	.487	0.01^e^
95% CI		[0.01; 0.03]	[0.02; 0.04]	[0.02; 0.04]			
Narrow BoS	10	0.04 (0.01)	0.04 (0.01)	0.04 (0.01)	0.086^a^	.918	0.12^e^
95% CI		[0.03; 0.04]	[0.03; 0.04]	[0.03; 0.04]			
Dual task	11	0.03 (0.01)	0.03 (0.01)	0.03 (0.01)	0.496^a^	.617	0.09^e^
95% CI		[0.03; 0.03]	[0.02; 0.03]	[0.02; 0.03]			
DTC (%)	11	−38.14 (36.90)	−32.86 (59.72)	−59.93 (127.26)	0.400^a^	.676	0.21^e^
95% CI		[−59.95; −16.34]	[−68.16; 2.43]	[−135.13; 15.28]			

*Note*. SD = standard deviation; *n* = sample size included in test; CI = confidence interval; BoS = base of support; DTC = dual task cost.

a ANOVA, *F*(2,20), Cohen's d.

b Friedman test.

c Post hoc test.

d Indicating comparison between T0 and T1.

e Indicating comparison between T1 and T2, χ^2^(2) *r*.

*P*-value all timepoints; *p < .05; **p < .1, trend.

All step activity data were normally distributed and analyzed using the paired two-sided *T*-Test. No significant changes were detected between the first and the second week of step activity measurement. The detailed values can be seen in Table 9. Noteworthy, though, the compliance rate of wearing the device for 7 consecutive days was 94.8% (74/77 days worn) in the first week and 83.1% (64/77 days worn) in the last week. One participant was excluded from the analysis as only two complete days were measured.

**Table 9.** Overview step activity monitoring outcomes.

	Mean (*SD*)					
Step activity indices	Week 1 (*n* = 11)	Week 2 (*n* = 10)	Mean difference (SD)	*T*(9)	*p*	*d*
Total steps (number)	3028.35 (921.18)	3338.03 (1298.64)	371.12 (1331.22)	0.882	.401	0.28
95% CI	[2483.97; 3572.72]	[2570.60; 4105.47]	[−415.57; 1157.81]			
Inactivity (minutes)	1192.27 (87.58)	1188.16 (95.22)	−14.46 (73.24)	−0.624	.548	0.20
95% CI	[1140.52; 1244.03]	[1131.89; 1244.43]	[−57.74; 28.82]			
Cadence (steps/minute)	13.24 (2.23)	14.90 (3.01)	1.37 (1.94)	2.234	.052**	0.71
95% CI	[11.92; 14.56]	[13.12; 16.68]	[0.22; 2.52]			
Low Intensity (minutes)	172.06 (79.33)	164.44 (80.30)	2.55 (41.85)	0.193	.852	0.06
95% CI	[125.18; 218.94]	[116.98; 211.90]	[−22.18; 27.28]			
Medium Intensity (minutes)	50.24 (14.67)	52.4 (24.01)	3.91 (24.28)	0.509	.623	0.16
95% CI	[41.57; 58.91]	[38.21; 66.59]	[−10.44; 18.26]			
High Intensity (minutes)	16.58 (10.41)	21.8 (10.37)	4.53 (9.41)	1.522	.162	0.48
95% CI	[10.43; 22.73]	[15.67; 27.93]	[−1.03; 10.09]			

*Note*. SD = standard deviation; *n* = sample size; CI = confidence interval; T(10), test statistic *T*-test (degrees of freedom); Effect sizes between T1 vs. T2: Cohen's *d*.

*P*-value between timepoints: *p < .05; **p < .1, trend.

All reaction time data were normally distributed except for the average reaction time of the steps to the right direction of the GoNogo Test, whereas all error data were non-normally distributed. The detailed outcome variable measures can be taken from Table 10. ANOVA detected a significant reaction time difference between timepoints in the Stroop Test [*F*(2, 20) = 3,748, *p *= .041*]. The Bonferroni *post hoc* correction showed a significant decrease (*p *= .022*) between T1 and T2 [1813.64 (*SD*: 436.51, 95% CI: 1555.68; 2071.60) ms versus 1597.51 (*SD*: 478.19, 95% CI: 1314.92; 1880.10) ms, respectively] with a large effect size (*d* 1.01). Moreover, ANOVA revealed a significant difference between time-points for the average reaction time of the steps to the left respective right direction [*F*(2, 20) = 7.865, *p *= .003*, *F*(2, 20) = 7.202, *p *= .004*, and *F*(2, 20) = 7.645, *p *= .003*, respectively] in the Reaction Time Test. With respect to the overall reaction times, *post hoc* tests revealed a significant decline (*p *= 0.030*) between T0 and T2 [1229.21 (*SD*: 115.27, 95% CI: 1161.09; 1297.33) ms versus 1077.44 (*SD*: 173.05, 95% CI: 975.18; 1179.70) ms, respectively] and a significant decline (*p *= .009*) between T1 and T2 [1259.70 (*SD*: 215.01, 95% CI: 1132.64; 1386.76) ms versus 1077.44 (*SD*: 173.05, 95% CI: 975.18; 1179.70) ms, respectively]. Effect sizes were large (*d* = 0.96 and *d *= 1.17, respectively). The Bonferroni correction showed a decline between T1 and T2 [1273.25 (*SD*: 241.48, 95% CI: 1130.54; 1415.95) ms versus 1083.18 (*SD*: 203.99, 95% CI: 962.63; 1203.73) ms, respectively] for the left side, which was significant (*p *= .009*) with a large effect size (*d *= 1.18). Regarding the right side, *post hoc* correction was able to show a significant decline (*p *= .012*) between T1 and T2 [1246.95 (SD: 194.32, 95% CI: 1132.11; 1361.78) ms vs. 1073.37 (*SD*: 146.80, 95% CI: 986.62; 1160.12) ms, respectively]. Hereby, the effect size was large as well (*d *= 1.11). No significance was detected in terms of the GoNogo Test overall reaction time or averaged reaction time to the left respective right side as well as for the Flexibility Test reaction times. Lastly, No significant differences were found for any assessment in terms of errors in the GoNogo Test.

**Table 10.** Overview cognitive-motor outcomes.

Cognitive-motor indices	Mean (SD)					Effect size
	Pre-T0	Pre-T1	Post-T2	F/χ^2^	*p*	
Overall RT (ms)						
Stroop test	1877.47 (485.43)	1813.64 (436.51)	1597.51 (478.19)	3.748^a^	.041*	1.01^d^
95% CI	[1590.60; 2164.34]	[1555.68; 2071.60]	[1314.92; 1880.10]		.022*^,^^c^^,^^d^	
GoNogo test	1018.79 (159.35)	1042.35 (167.65)	1015.14 (155.87)	0.372^a^	.694	0.24^d^
95% CI	[924.62; 1112.96]	[943.28; 1141.42]	[923.03; 1107.25]			
RTT	1229.21 (115.27)	1259.70 (215.01)	1077.44 (173.05)	7.865^a^	.003*	1.17^d^
95% CI	[1161.09; 1297.33]	[1132.64; 1386.76]	[975.18; 1179.70]		.009*^,^^c^^,^^d^	
Flexibility test	1631.42 (400.44)	1548.74 (379.14)	1483.16 (493.26)	0.977^a^	.394	0.18^d^
95% CI	[1394.78; 1868.06]	[1324.69; 1772.79]	[1191.67; 1774.66]			
Single RT (ms)						
GoNogo left	1026.98 (173.73)	1035.47 (190.96)	1016.67 (181.29)	0.127^a^	.881	0.18^d^
95% CI	[924.32; 1129.65]	[922.62; 1148.32]	[909.54; 1123.80]			
GoNogo right	1010.40 (153.46)	1051.88 (154.71)	1014.33 (147.66)	1.636^b^	.441	0.32^d^
95% CI	[919.72; 1101.09]	[960.45; 1143.30]	[927.08; 1101.59]			
RTT left	1247.02 (119.86)	1273.25 (241.48)	1083.18 (203.99)	7.202^a^	.004*	1.18^d^
95% CI	[1176.19; 1317.85]	[1130.54; 1415.95]	[962.63; 1203.73]		.009*^,^^c^^,^^d^	
RTT right	1215.18 (121.14)	1246.95 (194.32)	1073.37 (146.80)	7.645^a^	.003*	1.11^d^
95% CI	[1143.59; 1286.77]	[1132.11; 1361.78]	[986.62; 1160.12]		.012*^,^^c^^,^^d^	
Errors (number)						
Stroop test	12.09 (9.38)	8.73 (5.87)	7.18 (4.33)	2.053^b^	.358	0.40^d^
95% CI	[6.55; 17.63]	[5.26; 12.19]	[4.62; 9.74]			
GoNogo test	3 (4.36)	2.45 (5.89)	1.18 (2.99)	5.214^b^	.074**	0.42^d^
95% CI	[0.42; 5.58]	[−1.03; 5.93]	[−0.59; 2.95]			
RTT	0.82 (1.17)	0.55 (0.93)	0.36 (0.67)	1.826^b^	.401	0.25^d^
95% CI	[0.13; 1.51]	[−0.01; 1.10]	[−0.03; 0.76]			
Flexibility test	2.64 (2.20)	2.55 (2.30)	2.45 (3.08)	0.205^b^	.903	0.06^d^
95% CI	[1.33; 3.94]	[1.19; 3.90]	[0.64; 4.27]			

*Note*. SD = standard deviation; *n* = sample size included in test; RT = reaction time; RTT = reaction time test.

a ANOVA, *F*(2, 20), Cohen's *d.*

b Friedman test.

c Post hoc test.

d Indicating comparison between T1 and T2, χ^2^(2), *r.*

*P*-value all timepoints; *p < .05; **p < .1, trend.

**Figure 5.**
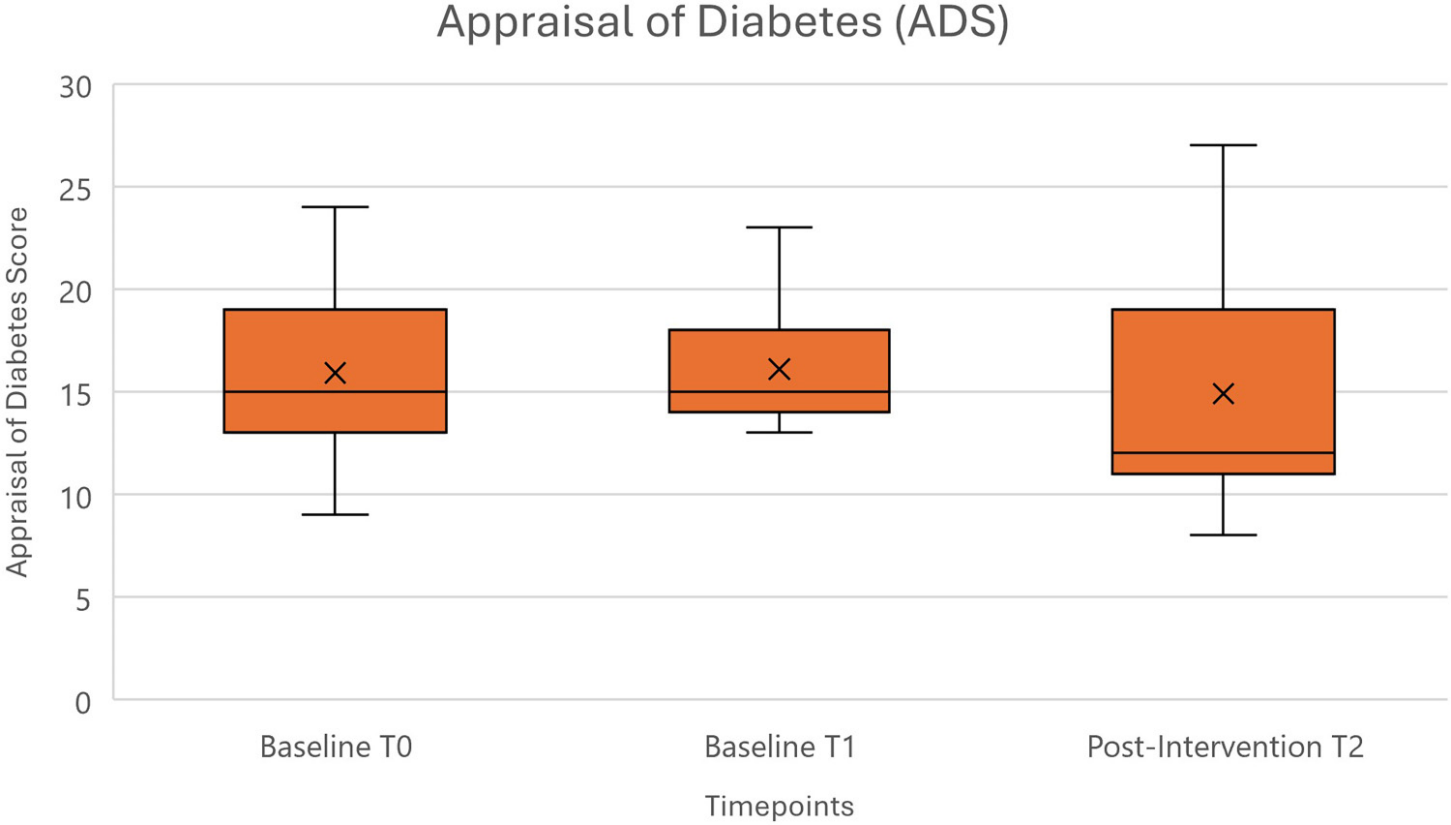
Appraisal of the diabetes scale (ADS) score means with standard deviations for all three measurement time points (*n* = 11).^52^

PRO data was normally distributed. No significant difference was detected by ANOVA [*F*(2, 20) = 0.562, *p *= .579]. The visualized comparison can be seen in Figure 5.

### PREMs

All participants took part in the interviews (*n* = 11), which lasted between 10 and 25 min. Conversations were impacted by language barriers because most participants had a migration background and had difficulties expressing complicated issues in German. Therefore, questions were asked using simple words and sentence structure. Participants’ statements were repeated by the interviewer to make sure that she understood what the participant meant. Consequently, interview data consisted primarily of snippets and unfinished sentences rather than comprehensive descriptions of the experience. The analysis resulted in two main categories: Reasons to participate and program experience and benefits, as shown in Table 11.

**Table 11.** Overview of categories used for the qualitative analysis.

Categories	Subcategories
Reasons to participate	Curiosity Participating in a fitness programme Improving existing impairments
Programme experience and benefits	Hard but doable Influence of trainer Effects on physical and mental well-being

#### Reasons to participate

Participants were recruited by their healthcare professionals during a consultation at the diabetes clinic. Their reason for participating in the program could be summarized in three subcategories: Curiosity, participating in a fitness program and improving existing impairments.

Curiosity: Participants were often just curious about the program. They felt as if they could participate in something new and interesting to them. For a few, curiosity was generated by their having nothing else to do.

My physician gave me the study information, so I thought that is interesting and exciting. (7)

… I am a curious person; I am always interested in new things. (12)

I was curious…and I have nothing to do, I am retired, I thought I do that then. (3)

Participating in a fitness program: Regular physical activity can be problematic for persons suffering from diabetes. This also proved true for most of our participants. The program gave them a structured and supported opportunity to be active. For those whose diabetes was well managed, it was an opportunity to do something preventive for aging.

I needed support in engaging in physical activity. Sport is a real problem for me. Then I became aware of the study, and I thought I do that now. You know I am not the person who goes in a fitness center… (7)

With relation to age, this is a good thing. One should engage more in fitness activities as one ages. (2)

Improving existing impairments: Dealing with complications of diabetes is quite challenging for those affected. Participants suffered from cognitive impairment, such as an inability to concentrate, and memory loss, as well as neurological and physical difficulties, such as diminished coordination and mobility, affecting their activities of daily living. Participants hoped that taking part in the program would help improve their impairments.

When I was asked by my health professional I thought, well I am diabetic; I have disorders, problems concentrating. Therefore, that program will be brain training and physical training for me. (p1)

Diabetes is a huge problem for me, for my whole family…so participating is something I do for it. (13)

#### Programme experience and benefits

The overall experience of the program was rated positively which is reflected within the following three subcategories: hard but doable, influence of trainer and effects on physical and mental well-being.

Hard but doable: The program proved to be challenging and a steep learning curve for those with existing impairments due to diabetes. Especially in the beginning, the program was strenuous and required high levels of concentration to master the games. They described “ups and downs” due to the varied complexity of the games, but after a few training sessions and getting used to the games their performance improved. Despite the challenges, all participants agreed to have had a positive experience and fun.

So, at the beginning it was difficult for me to realize the points (on the screen) in my head, I mean at first. Then it became easier… Sometimes it was easy, sometimes it was hard… (1)

At the beginning I didn’t understand it so well, but slowly I learned, I always learned, and it went well. (10)

Influence of Trainer: The trainer had a great influence on the positive experience of the participants during the program. Each participant appreciated his competence, friendliness, and patience during the sessions very much.

He explains different things and which part is what and when I don't understand something, he explains it to me in our language. I'm happy with Mr (name)… (6)

Um, that was great, absolutely. Well, I've rarely seen such a pleasant person who is so focused on you and supports you…he was always there during training, talking, joining in, participating. I found it extremely exciting and extremely good. So, I felt very, very comfortable during this whole time. (7)

They highlighted how well they felt supported, especially if they had some difficulty completing the games.

As for questions, they have always been there to be helpful. When I had difficulties, they were there. For example, if I don’t understand correctly, he has no problem saying it the second or third time. (11)

Effects on physical and mental well-being: According to most participants, the program had a positive influence on the physical and mental aspects of their well-being. Physically, they reported an improvement in mobility and body awareness. Cognitively, many participants experienced an improved ability to concentrate, an improved sense of balance as well as overall mental well-being.

Mobility, thinking and in the head. With therapy, everything is better. (3)

So, I went stand-up paddling once an in that time that sense of balance has gotten significantly better. (7)

Participating in the program motivated most of the participants to be more physically active in the future. For, some there was no notable difference. Participants with pre-existing cognitive impairment did not notice any difference in cognitive performance. The same was true for two men who were still working full-time.

Finally, despite all the challenges, everyone would recommend the program to others and most of them would consider continuing the program, if there was a mobile version they could use at home.

## Discussion

The present work aimed to design a versatile, motivating, and enjoyable training protocol on an existing exergame device and investigate its feasibility in a group of T2DM individuals. To the best of our knowledge, this is one of the first studies targeting executive functions in a T2DM population by implementing specific gaming tasks in an exergame training protocol beyond glycemic control. Concerning primary outcomes, almost all feasibility criteria were met within the preliminarily defined thresholds. Solely, the recruitment rate did not reach the specified threshold value of 20%. These results are in line with the existing literature on exergaming interventions and the elderly as well as with comparable studies in healthy elderly and diverse patient populations.^13,17,42,66,67^ The main reasons for not participating in the study were ‘not interested’, ‘no time’, and ‘long travel distance’ (see Study Flow Chart). The two latter are attributable for 35% of the exclusions. This exactly reflects the preferences of older people described by a paper stating that the type of exercise, travel time and costs are more highly valued than possible health benefits. In concrete, the preferences lie in at- or close-to-home solutions without any costs.^68^ The high adherence and compliance rates could be explained by the provided program characteristics such as the ubiquitous supervision, the protocol diversity with a wide selection of games, the self-determinable training frequency, and the short length of the training intervention period.^69^ Even though overall motivation and satisfaction were high, the exergame-based protocol was not able to significantly increase motivation. This aligns with recent findings from other feasibility and pilot studies investigating the feasibility of an exergaming taxonomy in chronic stroke patients as well as an active video game program for people with T2DM, respectively.^67,70^ However, this is in contrast with the existing literature stating that exergames are expected to increase self-determination and motivation.^20,67,71^ Though, the exergaming device displayed achieved points and the progress made over time in addition to motivational quotes like “Very good! Your performance has improved since the last time!” or “Keep up!,” the needs for competence, autonomy, and social relatedness were possibly not efficiently met.^71^ Yet, the technology was overall universally accepted with a remarkably high attitude towards using as well as a high behavioral intention to use and perceived usefulness, indicating a high motivation to continue the protocol and implement it in their lifestyle. This is supported by the qualitative data, as all participants would recommend the program to others and continue participation if there would be a mobile version. The high technology acceptance perfectly aligns with the current evidence.^42,66,72^ No participant experienced pain, dizziness, or any other adverse event related to the intervention. As mentioned above all adverse events were not related to the program, which perfectly agrees with a recent study assessing the feasibility of an exergaming intervention on the Dividat Senso also in terms of adverse event counts. Likewise, the authors reported no adverse events during training or measurements.^73^ Furthermore, this is in line with the general statement that exergaming is feasible and safe with minor adverse events.^66^

Secondarily, the present work aimed to investigate the possible effects of the specifically designed exergame training program on HRV, gait, step activity, cognitive-motor functions, and appraisal of diabetes. The corresponding results are discussed in the following sections in the respective order. The LF/HF ratio of HRV showed a medium effect size between T1 and T2. However, two of the cited studies used much longer intervention times (26 weeks and 24 weeks) and one of them investigated the effects of exergaming on HRV in a female group with fibromyalgia.^6^ Although six weeks of intervention duration were sufficient to increase HRV values using an exergaming-based dance training with short-duration high-intensity bouts, possibly, besides the low number of participants, the time frame was too short or the intensity too low to induce any changes in this case.^74^ In a more comparable setting, no differences were found in any of the HRV indices, even though the training consisted of high-intensity interval training (HIIT), which aligns with the present findings.^42^ It can be argued, that higher HRV values do not necessarily translate into better physical or cognitive performances for all activities.^75^ However, it has been shown that lowered HRV is mainly associated with impaired executive functions.^6^

Evaluating the effects on gait parameters, the pre- and postintervention results show negative changes in terms of variability in the narrow base of support as well as cycle duration and cadence in normal speed with large and medium-to-large effect sizes, respectively. Even though no significance was detected, positive changes were observed in stride velocity, stance and swing phase, and double support during the fastest speed testing. Thereby, the corresponding effect sizes were large and medium, respectively. These results are partially congruent with the findings from other studies conducting gait analysis to investigate the effects of exergaming on gait.^67,76^ There is, however, an accumulated body of evidence demonstrating the ability of exergaming to improve diverse spatio-temporal gait parameters in dual- and/or single-task conditions.^73,77–80^ the intervention's duration, frequency and the intensity may be factors that influenced the results. Even though the mean training intensity was moderate-to-high, the subjective ratings ranged from 1 to 9. It can be postulated, that some participants maybe did not train in their optimal zone, even though, according to the PREMs, the training was generally perceived as “*hard, but doable.*”

Exercise interventions using pedometers tend to increase daily step count by approximately 2000–2500 steps/day, following meta-analyses.^81–83^ Additionally, exergaming is generally able to increase physical activity.^17^ Most participants reported in the PREMs, that they were motivated to be more physically active throughout the day. However, no significant changes between timepoints were detected in any of the physical activity parameters measured by the step activity monitor. Solely, the overall cadence (steps/min) was approaching significance with a medium-to-large effect size. These results are in line with other studies showing no significant differences in the total step count.^84,85^ The presented data should be, however, interpreted with caution as the compliance rate was lower in the second week compared to the first. Beyond this, one participant had to be excluded from the analysis due to only two complete days measured and thereby missed requirements of at least three complete days per week.^85^ Hence, a well-established strategy for maintaining the compliance levels high should be taken into consideration to improve the comparability of the variables.

Exergaming interventions are capable of eliciting effects on cognition such as executive functions in diverse chronic disease populations as well as in healthy older adults.^73,86,87^ In agreement with the preliminary results from these studies, the presented exergame-based training protocol was able to affect some cognitive functions. The mentioned effects were limited to verbal inhibition and psychomotor speed. Most importantly, a significant overall decrease in reaction time was detected in the Stroop Test and the Reaction Time Test with large effect sizes. In the latter, both the average reaction time for the left side as well as for the right side decreased significantly between pre- and post-intervention time-points with large effect sizes. In addition, right side average reaction time in the GoNogo Test showed a medium effect size, indicating a positive effect of a six-week exergame training. To date, no exergame studies are currently available to compare the results of our study in participants with T2DM.

As stated by systematic reviews with meta-analyses, exergames can improve PROs in terms of health-related quality of life (HRQoL).^83,88^ Interestingly, some aspects of quality of life seemed to improve according to the qualitative analysis. Nevertheless, by using the diabetes-specific ADS questionnaire no meaningful changes were found over time in PROs. However, the overall literature is still inconclusive about the effects of exergaming on HRQoL, due to large heterogeneity and small sample sizes.^89^ Finally, further large-scale studies are needed to strengthen all the findings discussed above and to explore the possible effects of exergame training functional and patient-reported outcomes in T2DM in detail.

## Strengths, limitations, and future research

The number of assessments and the combination of motor and cognitive outcomes can definitely be counted as the strengths of the present work besides the valid and reliable measurement tools and the pre-defined feasibility protocol. The qualitative PREMs add a specific value to this study, with respect to the “user experience,” by providing insight into participants’ subjective perceptions.

This study was also affected by some limitations. At first, it must be kept in mind that the trial was designed to assess the feasibility of the exergaming protocol with a primary focus and not for evaluating its effectiveness. The context of this justification is that future studies will use the information from this trial in their design. Therefore, the sample size was small and furthermore, the target range of participants was not reached due to two dropouts. Consequently, the data should be interpreted with caution. Further, in the next step, when designing a randomized study, the recruitment procedure should be improved, for both glucose and neurocognitive measures in advance to determine an appropriate sample size.

Secondly, the sample showed a high variability in baseline values and was rather high functioning in mobility and cognitive assessments. Possibly this could explain the minor differences in the secondary outcomes.^90^ As a reason for that, it could be mentioned that the inclusion and exclusion criteria were defined very tight. In more detail, as seen in the flow chart (see Figure 2) depression or psychiatric disorder contributed to a total of 30 excluded candidates. According to an article in 2015, depression is present in one of every four individuals with T2DM.^91^ This restriction accounts for a hampered recruitment process and a lower recruitment rate as well as for recruiting participants with better initial values.

Thirdly, data assessed by measures like the MoCA, the SSK-scale, or the ADS should be interpreted cautiously, as they were performed in German only. A considerable proportion of the participants were not natives and therefore, their understanding of German was possibly not always sufficient to understand the tasks and questions. The MoCA for example is proposed to be performed in the mother language in order to deliver reliable outcomes.^92^ Finally, due to the nature of the study, the participants were instructed not to change their lifestyle and behavior during the first six weeks without training. Although, this was not controlled. This could also account for the variability between the two baseline measurements. The present project focused primarily on the feasibility and secondarily on functional and patient-reported outcomes in Diabetes mellitus type 2 patients. Therefore, in this early stage of research, no control for biasing factors is provided. In later stages and larger studies evaluating effectiveness as a primary outcome, controlling for biases as time since diagnosis, gender differences, glycemic control, carbohydrate metabolism, the dietary regimes and medical therapies and others should be implemented to present the whole picture of the effects of exergaming on functional outcomes.

As part of a multiple-step research plan, this study provides informative insights into several feasibility outcomes, building the foundation for the following projects. Further steps will aim for in-home solutions using the established training protocol. This might contribute to higher recruitment values and even higher adherence rates than observed. The program participation would, potentially, be more attractive from the beginning and frequencies higher than 2–3 times per week would be a valuable option. The following projects should take into consideration to implement high scores or leaderboards as well as live HRV biofeedback with the intention of personalizing training and exercising in the optimal zone. Randomized controlled trials with adequate sample sizes are warranted accompanied by accurate high-quality research study designs investigating the possible effects of this protocol on functional outcomes and PROs.

## Conclusion

In conclusion, the technology-based exergame training program specifically adjusted for individuals with T2DM is feasible with minor changes to the recruitment strategy and results in high motivation and adherence rates. Importantly, this trial delivers the fundamental knowledge for identifying effective gaming components and exergame training intervention design. Furthermore, the secondary results provide sound methodology with appropriate assessments, which might be useful for performing and reporting clinical trials with adequate samples in patients with T2DM.
